# The age-specific incidence of hospitalized paediatric malaria in Uganda

**DOI:** 10.1186/s12879-020-05215-z

**Published:** 2020-07-13

**Authors:** Arthur Mpimbaza, Richard Walemwa, James Kapisi, Asadu Sserwanga, Jane Frances Namuganga, Yasin Kisambira, Abner Tagoola, Jane Frances Nanteza, Damain Rutazaana, Sarah G. Staedke, Grant Dorsey, Jimmy Opigo, Alice Kamau, Robert W. Snow

**Affiliations:** 1 Child Health and Development Centre, College of Health Sciences, Makerere University, Kampala, Uganda; 2 Infectious Diseases Research Collaboration, Kampala, Uganda; 3 Department of Prevention, Care and Treatment, Infectious Diseases Institute, Kampala, Uganda; 4 Jinja Regional Referral, Hospital, Republic of Uganda Ministry of Health, Jinja, Uganda; 5 Mubende Regional Referral, Hospital, Republic of Uganda Ministry of Health, Mubende, Uganda; 6 National Malaria Control Program, Ministry of Health Uganda, Kampala, Uganda; 7 London School of Hygiene and Tropical Medicine, London, UK; 8 Department of Medicine, San Francisco General Hospital, University of California San Francisco, San Francisco, USA; 9 Population Health Unit, Kenya Medical Research Institute/Wellcome Trust Research Programme, Nairobi, Kenya; 10 Nuffield Department of Medicine, Centre for Tropical Medicine and Global Health, University of Oxford, Oxford, UK

**Keywords:** Age, Incidence, Hospitalized, Malaria, Uganda

## Abstract

**Background:**

Understanding the relationship between malaria infection risk and disease outcomes represents a fundamental component of morbidity and mortality burden estimations. Contemporary data on severe malaria risks among populations of different parasite exposures are scarce. Using surveillance data, we compared rates of paediatric malaria hospitalisation in areas of varying parasite exposure levels.

**Methods:**

Surveillance data at five public hospitals; Jinja, Mubende, Kabale, Tororo, and Apac were assembled among admissions aged 1 month to 14 years between 2017 and 2018. The address of each admission was used to define a local catchment population where national census data was used to define person-year-exposure to risk. Within each catchment, historical infection prevalence was assembled from previously published data and current infection prevalence defined using 33 population-based school surveys among 3400 children. Poisson regression was used to compute the overall and site-specific incidences with 95% confidence intervals.

**Results:**

Both current and historical *Plasmodium falciparum* prevalence varied across the five sites. Current prevalence ranged from < 1% in Kabale to 54% in Apac. Overall, the malaria admission incidence rate (IR) was 7.3 per 1000 person years among children aged 1 month to 14 years of age (95% CI: 7.0, 7.7). The lowest rate was described at Kabale (IR = 0.3; 95 CI: 0.1, 0.6) and highest at Apac (IR = 20.3; 95 CI: 18.9, 21.8). There was a correlation between IR across the five sites and the current parasite prevalence in school children, though findings were not statistically significant. Across all sites, except Kabale, malaria admissions were concentrated among young children, 74% were under 5 years. The median age of malaria admissions at Kabale hospital was 40 months (IQR 20, 72), and at Apac hospital was 36 months (IQR 18, 69). Overall, severe anaemia (7.6%) was the most common presentation and unconsciousness (1.8%) the least common.

**Conclusion:**

Malaria hospitalisation rates remain high in Uganda particularly among young children. The incidence of hospitalized malaria in different locations in Uganda appears to be influenced by past parasite exposure, immune acquisition, and current risks of infection. Interruption of transmission through vector control could influence age-specific severe malaria risk.

## Background

Despite reductions in the risk of malaria infection, morbidity and mortality across sub-Saharan Africa (SSA) over the last two decades, gains have not been uniform in the region [1, 2]. Six countries account for nearly half of the world’s malaria disease burden including Nigeria (25%), Democratic Republic of the Congo (12%), Uganda (5%), and Cote d’Ivoire, Mozambique and Niger (4% each) [2]. The latest figures for Africa suggest that life-threatening morbidity leading to death from malaria across Africa remains unacceptably high [2].

During the 1990s and early 2000s the relationship between the frequency of malaria parasite exposure and severe disease incidence was the subject of intense investigation across different transmission settings in African [3–12]. These studies formed the basis of provisional modelling of the relationship between infection exposure and severe disease outcomes [13–16]. The likelihood of a new malaria infection resulting in life-threatening disease is principally determined by the ability of the host to mount an immune response; a function of previous parasite exposure, age and prompt treatment of mild disease with effective medicines. The relationship between parasite exposure and disease outcome represents the fundamental basis of the determination of the malaria disease burden in time and place [17–19]. Over the last 15 years there have been few investigations on the clinical epidemiology of severe malaria, notably during a time of changing parasite transmission intensity and treatment access across SSA, with research into severe malaria becoming a neglected topic [20].

In Uganda, despite a reduction in national under-five malaria prevalence from 30.4% in 2016 [21] to 16.9% in 2018–2019 [22], modelled projections, show that the country experienced approximately 12.3 million malaria cases and 13,203 malaria deaths in 2018, with little evidence of change since 2016 [2]. Malaria continues to contribute to 20–30% of all paediatric admissions to hospitals across the country [23], overwhelming emergency clinical services. The last comparisons of the rates and clinical phenotypes of hospitalized malaria, at three sites in Uganda, was undertaken in 2002–2003 [7]. Almost 20 years on, the link between a history of infection and severe disease is poorly defined, limiting our ability to understand the effects of infection prevention through vector control on disease profile in Uganda. Here we present a comparison of the rates of malaria hospitalisation and the clinical spectrum of severe disease across five catchment areas close to district hospitals where the historical and current malaria parasite exposure has been characterized.

## Methods

### Hospital surveillance

In 2010, the Uganda Malaria Surveillance Project (UMSP), in collaboration with the National Malaria Control Programme (NMCP), established an inpatient malaria surveillance program in the children’s wards at six public hospitals [24–27]. These six hospitals reflected the diversity of malaria transmission across Uganda. A structured Medical Record Form (MRF) was developed, enabling a standardized information record system for all admissions at participating sites. The MRF facilitated capture of detailed individual level information including patient demographics, residential address, presenting symptoms and clinical signs, laboratory test results, treatment prescribed, diagnosis and outcome upon discharge. Attending nurses, clinical officers, and physicians completed the MRF. UMSP supplied MRFs and basic malaria laboratory supplies. Health workers were trained on the use of the MRF, importance of good medical record keeping, use of data to improve the quality of services, and proper case management of uncomplicated and severe malaria. Emphasis was placed on ensuring all children were screened for malaria parasites using microscopy and/or malaria Rapid Diagnostic Tests (mRDT) on admission or during admission. Haemoglobin concentrations were only performed when requested by clinicians.

In 2016, the inpatient malaria surveillance program was expanded to provide a platform for investigation of aetiologies of Acute Febrile Illnesses (AFI) [27]. Improvements were made to the coverage of routine malaria testing and the documentation of patient’s physical address. The MRF was adapted to incorporate additional variables relevant to expanded bacterial and viral diagnostic capacity, described elsewhere [27]. Under the AFI program, the data management system was upgraded to a web-based data management system hosted on the District Health Information System (DHIS-2), allowing for real time data entry.

**Fig. 1:**
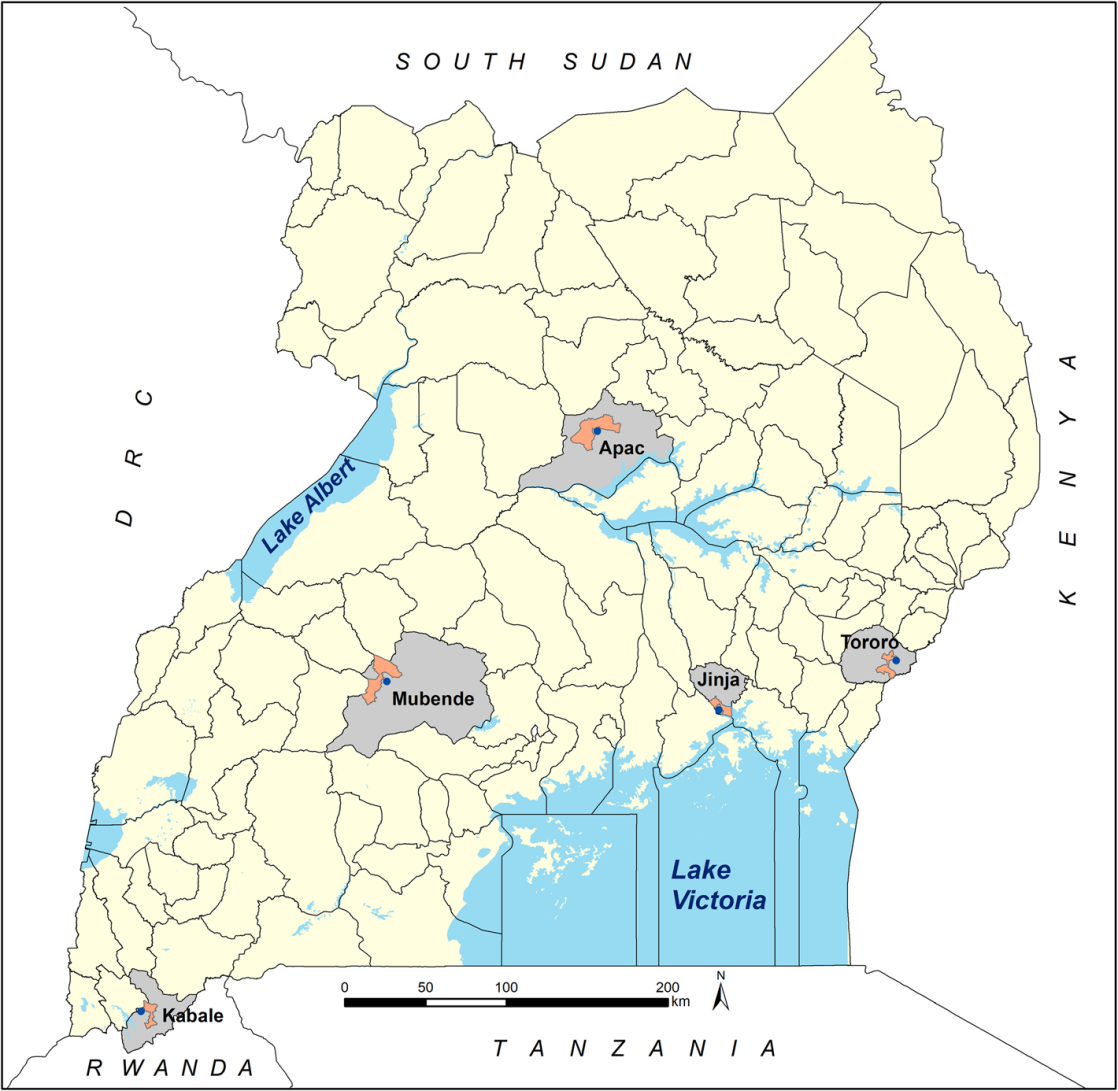
Location of five sentinel districts (grey) and parish catchment areas used to define malaria hospitalisation rates (orange) at select hospitals (blue dots)

The present study focused on a retrospective analysis of admissions to five hospitals from 2017 to 2018 among children aged 1 month to 14 years from communities located close to five hospitals shown in Fig. 1 and Table 1. Arua hospital was not included in this analysis because it served a large refugee community hence might not have represented a stable population subject to local malaria exposure.

**Table 1:** Hospital and catchment characteristics

Characteristics	Apac District Hospital	Tororo District Hospital	Mubende Regional Referral Hospital	Jinja Regional Referral Hospital	Kabale Regional Referral Hospital
Surveillance period (months)^a^	Apr 2017-Dec 2018 (21 months)	Apr 2017-Dec 2018 (21 months)	Jan 2017-Dec 2018 (24 months)	Jan 2017-Dec 2018 (24 months)	Mar 2017-Dec 2018 (22 months)
District, Region	Apac, Lango	Tororo, Bukedi	Mubende, North Buganda	Jinja, Busoga	Kabale, Kigezi
Altitude, meters above sea level	1054	1220	1314	1182	1862
Average annual rainfall during surveillance period (mm/year)	1587	1474	1062	1445	1050
HbAS frequency [28]	19.2	19.6%	10.9%	19·5%	2.6%
District parasite prevalence 2010–2016, (number of surveys [number individuals examined]^b^	42.0% (26) [917]	56.3% (105) [38078]	22.9% (2) [48]	41.4% (45) [3112]	4.9% (3) [122]
History (dates) of LLIN mass distribution [23]	May 2014, Feb 2017	May 2017	Nov 2017	Mar 2017	May 2014, Jun 2017
History (dates) of IRS campaign [23]	Mar 2008 DDT Feb 2010 Alpha-cypermethrin Aug 2010 Bendiocarb Jan, May, Nov 2011 Bendiocarb Apr, Oct 2012 Bendiocarb Apr, Nov 2013 Bendiocarb Apr 2014 Bendiocarb May 2017 Actellic	Dec 2014 Bendiocarb Jun, Nov 2015 Bendiocarb Jun 2016 Actellic Jul 2017 Actellic Jun 2018 Actellic	None	None	Dec 2006 lambda-cyhalothrin Mar 2007 lambda-cyhalothrin
Total admissions during surveillance period	4931	7122	4410	13,320	2314
Total malaria admissions during surveillance period	2815	1840	1870	3692	28
Catchment Parishes	Atik, Amii Abedi, Atopi	Osia, Osukuru Nyakesi, Nyangole	Kabowa, Katente Kibalinga	Buwende, Kimaka, Mafubira, Masese, Walukuba East&West	Bugarama, Kahoro Kigata, Muyumbu, Nyakagyera
Minimum-maximum distance to hospital (km)	2.3–16.3	2.0–11.5	3.4–18.6	1.3–9.0	3.9–11.7
Census projected population years at risk 2017–18 aged 1 month to < 15 years	37,680	40,852	48,701	82,671	22,504
**School survey results within selected parishes**					
Number of schools/number of pupils 6–16 years	6/601	7/824	4/403	8/800	7/800
*P. falciparum* parasite prevalence (microscopy)	58.2%	7.5%	32.2%	10.2%	0.1%
Use of LLIN night before survey	23.7%	41.6%	43.5%	54.3%	89.2%
IRS reported in child’s household in last 12 months	6.6%	97.4%	1.2%	5.5%	0.7%

^a^Surveillance for AFI began at different times at each of the five hospitals

^b^Survey data from district assembled and described in ref [1], available at [29] and updated with additional data (see Methods), used here in covering 2010–2016 to represent parasite exposure in the district prior to the surveillance period. School survey data represent a more location-specific estimate of infection prevalence at the end of the hospital surveillance period (2019)

### Case definitions

Patient diagnoses were made at admission and at discharge. A check list of most common diagnoses was incorporated in the MRF. Upon evaluation of patients, based on their judgment informed by history, examination findings and available test results, clinicians checked one or more diagnosis. It was not unusual for children to have more than one diagnosis; occasionally complications of illness were listed as diagnoses. A free text field was included for recording diagnosis not included in the list of available diagnosis. Patients who had any record under the possible diagnoses, or notes, of AIDS, TB, Sickle Cell Disease, epilepsy, congenital malformations, poisoning, burns, trauma, snake or animal bites, elective surgery or measles were immediately excluded from the data series, irrespective of whether they had a diagnosis of malaria. Here we presumed the underlying conditions to be the primary cause for admission and malaria a coincidental diagnosis. Patients who had any record of malaria were then identified and checked against malaria test (microscopy or mRDT) results, those who had a negative malaria test results were reclassified as unknown or replaced by other diagnoses if reported.

Malaria admissions were further classified according to the criteria of severe malaria approximating to those defined by the WHO [30] including, cerebral malaria, severe malaria anaemia, respiratory distress and haemoglobinuria, based on admission symptoms, signs, and laboratory findings. Consciousness was defined through observation of the child at admission. Unconsciousness was not formally evaluated using a Blantyre or Glasgow coma score, rather an APVU (Alert, responds to Pain or Voice or Unconscious) score was used [31], where U, approximates to a Blantyre Coma Score of 3 or less. Given the incomplete nature of haemoglobin results on all malaria admissions, it was not possible to use the strict definition of severe malaria anaemia (SMA) [30]. Therefore, SMA was classified if a) available haemoglobin results at, or during, admission was< 5 g/dl; b) for those without a haemoglobin result < 5.0 g/dl, if the child received blood transfusion during admission; and then c) if not classified by a) and b) whether they presented on admission with clinical signs of severe pallor. Respiratory distress was classified as deep breathing recorded through observation at admission. Haemoglobinuria was classified as present based on a reported history of passing ‘tea’ coloured urine by caretakers of the admitted child. Other features of severe malaria [30] could not be classified from information available on the MRFs, including prostration (age relevant inability to sit, stand or breastfeed not standardized between sites), hyper-parasitaemia (parasite counts per microlitre not recorded) or multiple convulsions within 24 h. Whether a child had had a seizure during the illness episode was documented, however, the number, type and duration of seizures was not recorded. The outcome of admission was incomplete across all hospitals, including large numbers having absconded prior to formal discharge, making case-fatality rates impossible to compute overall or for specific disease phenotypes. All relevant data were abstracted from the electronic MRF databases. All the subsequently selected patients aged 1 month to 14 years from the catchment areas with missing data in the electronic database were crosschecked against original hard copy clinical and laboratory registers for completeness. A set of unique identifiers were used to match electronic records to entries in the register.

### Defining residences and denominators

A variety of information on each patient’s residential address was available on the MRF. This information was used to locate residential parishes (third level census administration areas). Lists of village names per parish, Google Earth and other digital place name gazetteers [32] were used to provide a parish code for all the malaria admissions. Rural, or peri-urban, parishes were then selected that were closest, within 20 km, to the hospital. Urban residences were excluded, as anecdotal evidence suggests that they may have been provided by visitors from other areas rather than local residents. Twenty kilometers is within the distances used to define optimized hospital catchments in Tanzania [33], Uganda [34, 35] and Kenya [36–38], accounting for most acute febrile admissions. Furthermore, the selection of parishes close to the sentinel hospital excluded communities that may have had better access to “competing” hospitals elsewhere in respective regions.

The selected parishes were then used to extract total population counts from the 2014 national census [39], projected to 2018 using reported district intercensal growth rates. Total population counts were corrected to single year age group (0-, 1-, 2- … 14 years) denominators using regional rural, household census data from the 2016 Demographic and Health Survey in each respective region [40]. Age-specific populations at risk were finally corrected to the number of person-years-of observation depending on the surveillance period, with the first year of life adjusted for 11 months.

### Defining transmission intensity

To understand the role of parasite exposure on the epidemiology of severe malaria risks both historical and current infection prevalence were defined. It was not possible to identify specific survey data on community-based malaria prevalence for each of the catchment areas selected for the present study. However, district-wide survey data undertaken between 2010 and 2016 among children aged less than 15 years, where at least 15 children had been examined with microscopy were identified from previous datasets [1, 29] updated with other sub-national survey data [41]. Data were aggregated as mean estimates of age-corrected (2–10 years) prevalence and crude estimates of prevalence (total positives/total examined) for all surveys 2010–2016 pre-hospital surveillance period.

To represent contemporary infection prevalence, school-based surveys within the catchment parishes selected for the 2017–2018 surveillance period were undertaken at the end of the hospital recruitment period in 2019. School children provide a simple, reliable source of community-level malaria prevalence [42, 43]. Catchment parishes were used to define sample size. An estimate of expected district prevalence was used from previously analysed malaria prevalence risk undertaken by the NMCP [44]. A precision for the estimate was set at 5% and the confidence interval set at 95%, and assuming a non-response rate of 10%. Based on these assumptions, a total of 3400 children in 33 schools participated in the survey. For each area, schools were randomly selected from district inventories of public, day schools, 100 to 150 children were randomly selected to participate from classes Primary One to Primary Six from each school in the region. Children of consenting parents were interviewed to collect information on demographics, mosquito net usage during the night prior to the survey, use of indoor residual house-spraying in their homesteads and recent history of illness including fever. A finger prick blood sample was collected for thick and thin blood smear and a malaria rapid diagnostic test (mRDT) (CareStart™) for detection of malaria parasites. Thick and thin blood smears were stained with 2% Giemsa for 30 min. 100 high power fields were used to detect and quantify infection using experienced UMSP microscopists. mRDT results were read as per manufacturer’s instructions. Children with positive mRDT results were treated with artemether-lumefantrine as per recommended national guidelines [45]. Slide results were considered final if the first and second expert readings agreed on parasites densities (< 25% level of disagreement). If the first and second expert readings were discordant, a third expert-reader was used to arbitrate.

### Analysis

All clinical data were stored on the DHIS-2 platform and school data were double entered using EpiData (v3.1). Data analysis was performed in STATA (version 14; STATA Corp., College Station, TX, USA) and R version 3.6.1 (R Core Team (2019), Vienna, Austria). Patient’s demographics were summarised as proportions and median ages with interquartile range (IQR). Kruskal Wallis was used to test for differences in median age. The incidence rate (IR) of hospitalised malaria was defined as the number of cases with a primary diagnosis of malaria (confirmed by microscopy or mRDT) divided by person-years of observation expressed per 1000 people. Since re-admissions were not documented all malaria admissions were included in the IR calculation, and therefore strictly best referred to as period prevalence. The prevalence of each severe malaria phenotype was defined as the number of hospitalised children with a specified severe malaria phenotype divided by the number of all hospitalised children without missing information required to classify by that phenotype. The age specific, site specific, and syndrome specific IR with 95% confidence intervals were computed using the exact Poisson distribution. Poisson regression was used to compute the incidence rate ratios (IRR) for the age groups < 1 year, 1–4 years, 5–9 years and 10–14 years, adjusted for site. The association between parasite prevalence and IR of malaria hospitalisation was determined using a Spearman rank correlation coefficient (rho) and confidence intervals constructed using Fisher’s z transformation.

## Results

### Characteristics of study sites

The five rural, catchment parishes used to define hospital admission rates are shown in Table 1 and Fig. 1. Surveillance at each of the five hospitals started at different times in 2017 and observation periods ranged between 21 and 24 months, ending in December 2018 (Table 1). The projected population years of observation for children aged 1 month to less than 15 years within each catchment area is shown in Table 1. Catchment areas were between 2 and 19 km from respective hospitals.

The five selected hospital sites (Fig. 1) and the districts where they are located, represent historically stable, endemic malaria ecologies as demonstrated by the high frequencies of the heterozygote state of the sickle cell gene (haemoglobin AS) and high average annual rainfall. The exception being the high-altitude district of Kabale; a low transmission setting (Table 1). Between 2010 and 2016, prior to the hospital surveillance, community-based childhood parasite prevalence in the five districts ranged from 4.9% in Kabale to 56.3% in Tororo. During this period the natural endemicity in Apac was altered using biannual rounds of indoor residual spraying (IRS) with Bendiocarb from 2010 to 2014 [46] (Table 1). IRS was suspended in Apac in May 2014 [47], however one round of IRS with Actellic® was conducted in May 2017 [48]. In Tororo, biannual IRS with Bendiocarb began in December 2014 through 2015, in 2016 IRS involved annual spray rounds with Actellic® [49] and continued throughout the hospital surveillance period 2017–2018 (Table 1). Vector control in Mubende, Kabale and Jinja has relied only on Long-Lasting Insecticide treated Net (LLIN) distribution.

The school-based surveys among children aged 6–16 years, undertaken at the end of the hospital surveillance period, demonstrated a diverse pattern of malaria endemicity ranging from < 1% *Plasmodium falciparum* slide positivity in Kabale to 54% in Apac (Table 1). These estimates of community-based infection prevalence reflect historical transmission ecology (Kabale) and recent suspension (Apac) or introduction (Tororo) of IRS. Furthermore, reported LLIN use the night prior to the survey by school children was lowest in Apac (24%), and between 40 and 90% in all other catchment areas (Table 1).

### Age and malaria admissions from selected parishes

One thousand seven hundred eight admissions aged 1 month to 14 years of age with a primary diagnosis of malaria, confirmed by microscopy and/or mRDT, without underlying causes for admission were identified with a residence within selected catchment areas across the five hospitals. The overall hospitalisation incidence rate (IR) across the combined sites was 7.3 (95% CI 7.0, 7.7) per 1000 children 1 month to 14 years per annum. The annualized rates of malaria hospitalisation varied significantly between hospital catchments, with the lowest rates observed at the very low transmission site at Kabale (IR = 0.3; 95% CI: 0.1, 0.6) and the highest at Apac (IR = 20.3; 95% CI: 18.9, 21.8) (Table 2).

**Table 2:** Measures of malaria morbidity in different age groups among children hospitalized with malaria at five public hospitals in Uganda

	Apac District Hospital	Tororo District Hospital	Mubende Regional Referral Hospital	Jinja Regional Referral Hospital	Kabale Regional Referral Hospital
**Overall (1 month - 14 years)**					
Person Years Observation at Risk	37,680	40,852	48,701	82,671	22,504
Median age in months (IQR)	36 (18, 69)	28 (17, 48)	30 (16, 54)	27 (18, 54)	40 (20,72)
Number of cases	764	317	253	368	6
Incidence Rate (95% CI)	20.3 (18.9, 21.8)	7.8 (6.9, 8.7)	5.2 (4.6, 5.9)	4.5 (4.0, 4.9)	0.3 (0.1, 0.6)
**< 1 year**					
Person Years Observation at Risk	1983	2670	3251	5469	1365
Number of cases	92	37	30	54	0
Incidence Rate (95% CI)	46.4 (37.4, 56.9)	13.9 (9.8, 19.1)	9.2 (6.2, 13.2)	9.9 (7.4, 12.9)	0 (0, 2.7)
**1–4 years**					
Person Years Observation at Risk	10,284	12,142	14,922	23,781	6537
Number of cases	436	213	164	228	3
Incidence Rate (95% CI)	42.4 (38.5, 46.6)	17.5 (15.3, 20.1)	11.0 (9.4, 12.8)	9.6 (8.4, 10.9)	0.5 (0.1, 1.3)
**5–9 years**					
Person Years Observation at Risk	13,124	13,685	16,650	28,285	7807
Number of cases	171	58	50	70	3
Incidence Rate (95% CI)	13.0 (11.1, 15.1)	4.2 (3.2, 5.5)	3.0 (2.2, 4.0)	2.5 (1.9, 3.1)	0.4 (0.1, 1.1)
**10–14 years**					
Person Years Observation at Risk	12,289	12,355	13,878	25,136	6793
Number of cases	65	9	9	16	0
Incidence Rate (95% CI)	5.3 (4.1, 6.7)	0.7 (0.3, 1.4)	0.6 (0.3, 1.2)	0.6 (0.4, 1.0)	0 (0, 0.5)

Incidence rate per 1000 children p.a

**Fig. 2:**
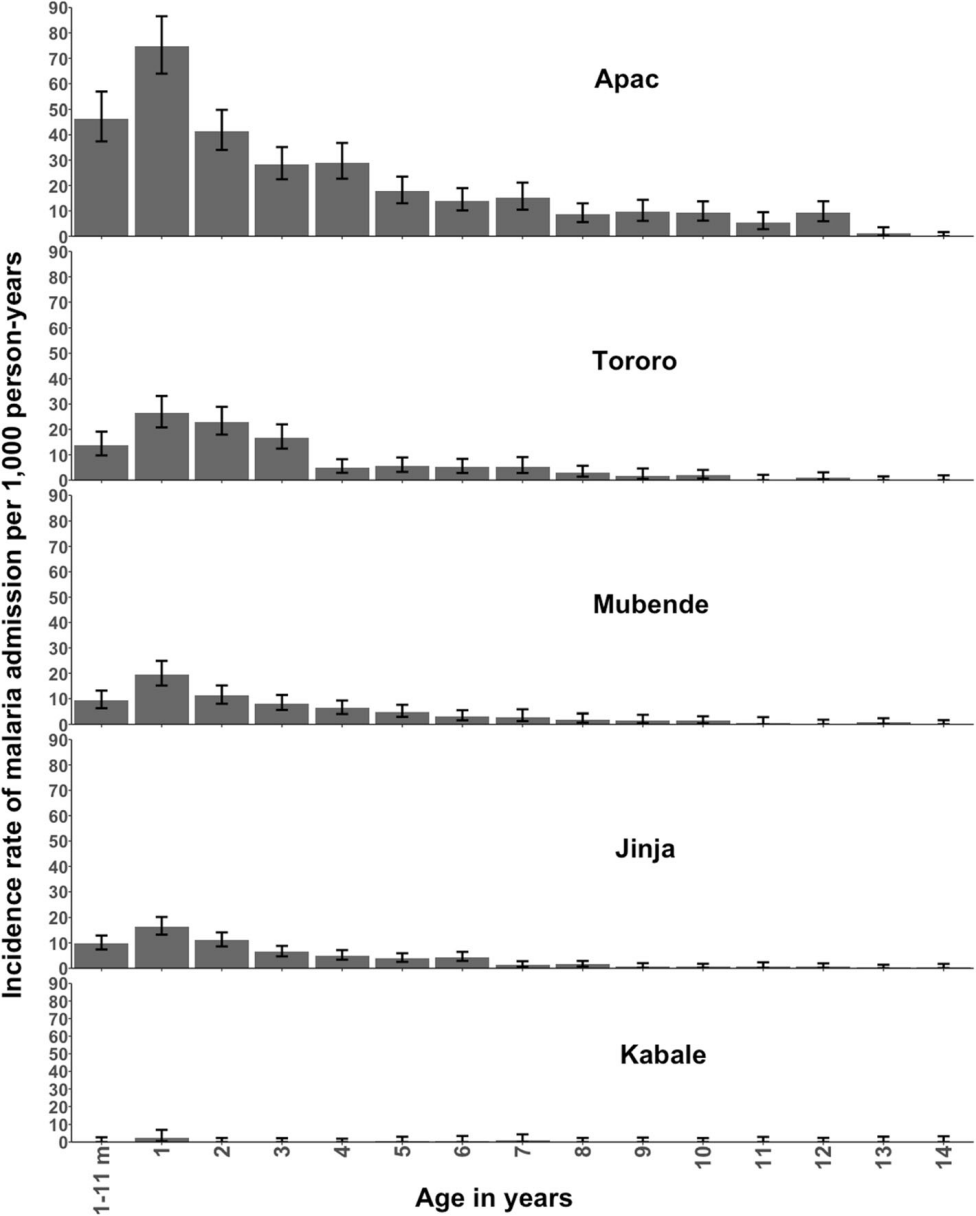
Age-specific malaria admission rates per 1000 person-years of observations at five sites (showing 95% confidence intervals)

Among all admissions the median age of malaria hospitalisation was 30 months (IQR: 17.5, 60), 1257 (73.6%) of all admissions were aged less than 5 years. Children presenting to Kabale hospital had the highest median age (40 months; IQR: 20,72), followed by Apac (36 months; IQR: 18, 69) and the remaining sites the median age of the malaria admissions was between 27 and 30 months (Table 2). Except for Kabale, rates of admission at each of the other four sites increased after the first birthday, peaking during the second year of life, and declining thereafter (Fig. 2). Rates of malaria hospitalisation were lower among children aged 5–9 years (IRR = 0.28; 95% CI: 0.25, 0.32; *p* < 0.001) and among those aged 10–14 years (IRR = 0.09; 95% CI: 0.07, 0.11) as compared to children aged 1–4 years after adjusting for site (Supplement S1).

**Fig. 3:**
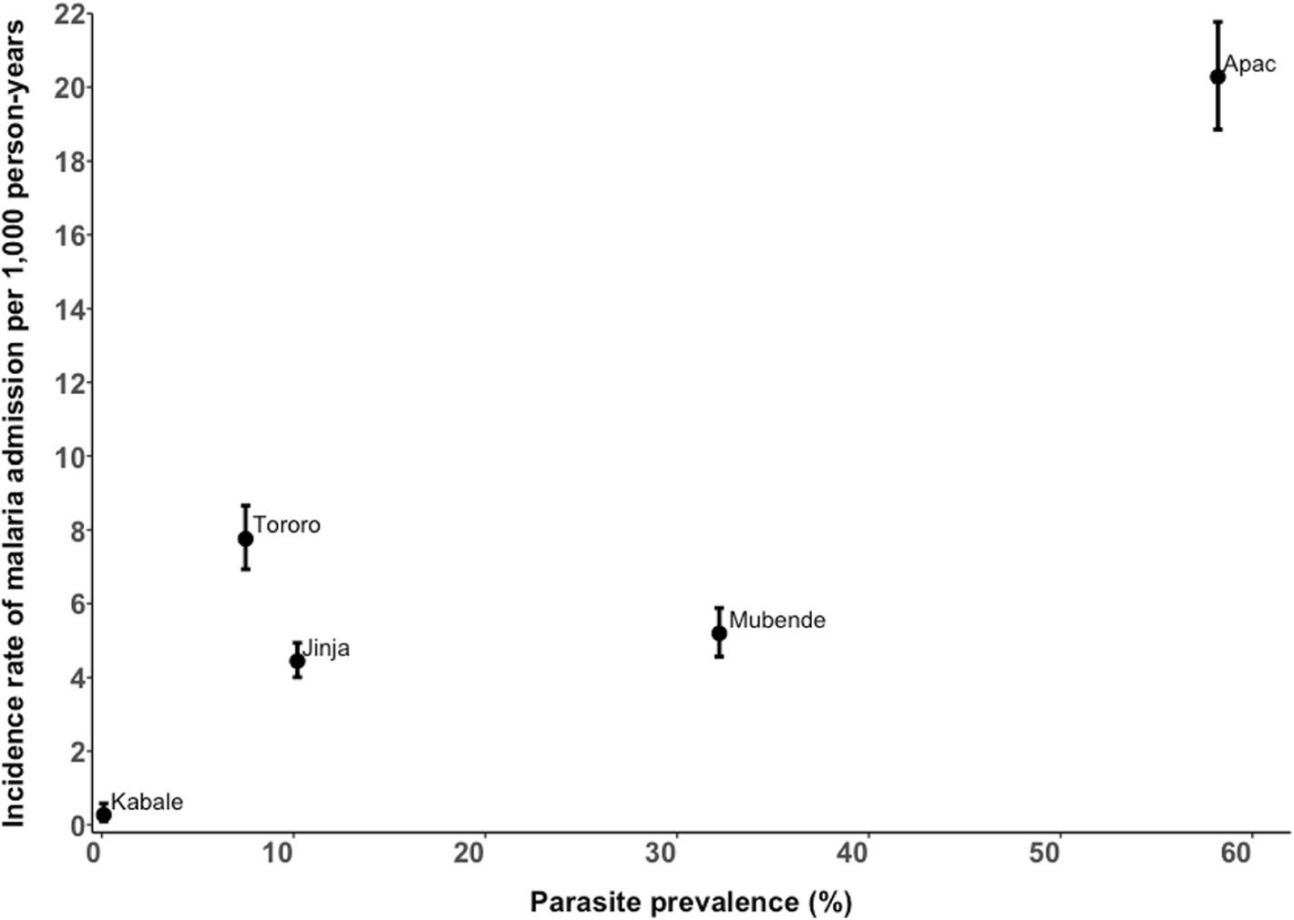
Malaria admission rates for children aged 1 month-14 years per 1000 person-years of observation versus school-based malaria parasite prevalence at the end of the surveillance period

There was correlation between current catchment-specific school-based infection parasite prevalence and the overall malaria admission incidence rate in each site (rho = 0.70; 95% CI: − 0.48, 0.98) (Fig. 3), although this did not reach statistical significance (*p* = 0.19). The association remained unchanged even when we restricted the rates of admission to children aged 1–23 months of age (rho = 0.70; 95% CI: − 0.48, 0.98; *p* = 0.19), a period most likely representative of current transmission intensity (Supplement S2).

### Prevalence of severe malaria phenotypes

Overall, unconsciousness (a surrogate marker of cerebral malaria, 30/1674; 1.8%) was the least common severe disease phenotype among all admissions. However, unconsciousness was present in 1/6 of the admissions at Kabale and was more common in Mubende (19/253; 7.5%) compared to other sites (Table 3). The composite definition of severe malaria anaemia (SMA) depended on the availability of information, a total of 129 (7.5%) SMA events were recorded, 37 based on haemoglobin, 63 based on a history of blood transfusion and 29 based on a presentation of severe pallor. Of all admissions, 34 (2%) and 10 (0.6%) patients could not be classified for unconsciousness and SMA, respectively, for lack of relevant information. Overall, the composite indicator of SMA (129/1690; 7.6%) was considerably more common than unconsciousness and most prevalent among malaria admissions at Mubende (51/253; 20.2%) and least prevalent in Apac (15/762; 2.0%) (Table 3). No children with SMA were noted in Kabale hospital. Evidence of respiratory distress was present among 55/169 (3.2%) malaria admissions, a history of passing ‘tea’ coloured urine (haemoglobinuria) was reported as present in 84/1691 (5%), and highest at Mubende 21/253 (8.3%) (Table 3). Overall, children who were unconscious (median age 60 months, IQR: 26, 84) were older than those who had severe anaemia (median age 36 months, IQR: 18, 72), respiratory distress (median age 19 months, IQR: 13, 42), and haematuria (median age 48 months, IQR: 23, 72), (*p* < 0.001) (Table 3).

**Table 3:** Prevalence of severe malaria phenotypes among resident children in defined catchments areas of specific hospitals

Severe malaria phenotype	All sites	Apac District Hospital	Tororo District Hospital	Mubende Regional Referral Hospital	Jinja Regional Referral Hospital	Kabale Regional Referral Hospital
**Unconscious**						
n/N (%)	30/1674 (1.8%)	4/753 (0.5%)	1/296 (0.3%)	19/253 (7.5%)	5/366 (1.4%)	1/6 (16.7%)
Median age in months (IQR)	60 (26, 84)	90 (45, 132)	18 (18, 18)	48 (24, 84)	70 (26, 96)	60 (60, 60)
**Respiratory distress**						
n/N (%)	55/1699 (3.2%)	14/762 (1.8%)	14/310 (4.5%)	18/253 (7.1%)	9/368 (2.5%)	0/6 (0%)
Median age in months (IQR)	19 (13, 42)	19 (14, 41)	14 (9, 66)	31 (15, 42)	15 (12, 26)	–
**Tea coloured urine**						
n/N (%)	84/1691 (5.0%)	43/761 (5.7%)	8/303 (2.6%)	21/253 (8.3%)	12/368 (3.3%)	0/6 (0%)
Median age in months (IQR)	48 (23, 72)	41 (18, 72)	32 (20, 42)	60 (48, 72)	63 (32, 90)	–
**SMA**						
n/N (%)	129/1690 (7.6%)	15/762 (2.0%)	21/307 (6.8%)	51/253 (20.2%)	42/368 (11.4%)	0/6 (0%)
Median age in months (IQR)	36 (18, 72)	70 (24, 108)	36 (24, 50)	36 (18, 60)	42 (18, 72)	–

*SMA* Severe malaria anaemia, described in text; the incidence rate is per 1000 children p.a., *n* The number of hospitalised children with a specified severe malaria phenotype, *N* The number of all hospitalised children without missing information required to classify by that phenotype

## Discussion

We have presented an ecological comparison of the rates of malaria hospitalisation against estimates of historical and current levels of malaria transmission from five communities in Uganda. Unsurprisingly, in areas of historical and contemporary very low transmission, paediatric malaria hospitalisation is uncommon (Kabale). Consistent with earlier observations in high transmission settings in Africa during the 1990s-early 2000s [3–12], Apac exhibited an extremely high incidence of hospitalized malaria among children aged under 24 months of age (Fig. 2). However, unlike previous reported age-prevalence and incidence estimates from areas with hyper-endemic transmission studied in the 1990s [3–12], Apac demonstrated an age-profile of hospitalized malaria incidence that included a much higher incidence among older children aged 5–10 years and 10–15 years (Table 2; Fig. 2). The unexpectedly high rate of malaria admissions among older (> 5 years) children in Apac, traditionally an area of intense transmission, may have resulted from the rapid reduction in malaria parasite exposure following the IRS campaigns in 2010–2014 [46]. The historical reduction in transmission may have facilitated the emergence of an ‘atypically’ non-immune cohort of older children in 2017–18 unusually susceptible to malaria and its complications. Periods of sustained malaria control, followed by relaxation or withdrawal of effective interventions, has been shown to result in rebound [50], which was also documented in Apac [47].

Since 2014, the traditionally high transmission (Table 1) region in Tororo, continues to be under intensive IRS (Table 1) [49], sustaining protection against infection among children aged 1 month to 4 years of age at-risk of hospitalisation with malaria during the 2017–2018 surveillance period. In Tororo children older than 5 years would have been exposed to high transmission intensity from birth (Table 1). Contemporary estimates of infection prevalence are 7.5% compared to an estimated historical prevalence above 50% (Table 1). The age pattern and broad overall rate of malaria hospitalisation were similar in Tororo compared to Jinja and Mubende, with current transmission between 10 and 32% respectively, with 74% of all paediatric admissions occurring before the 5th birthday and a peak in the second year of life (Table 2; Fig. 2). Despite a significant reduction in parasite exposure in Tororo, rates of malaria hospitalisation remain high compared to other sites of sustained high transmission (Mubende) or in transition without the use of IRS (Jinja). Infection risks have not been eliminated in Tororo and some premunition might still be occurring among young children. However, cessation of IRS might result in a changing age-profile of disease as witnessed in Apac.

Overall, cerebral malaria was comparatively uncommon as a severe complication of malaria admissions and when observed had a higher mean age compared to other severe disease phenotypes (Table 3). This has been recently confirmed in Kenya [31]. Severe malaria anaemia (SMA), as defined in our series based on available information on haemoglobin concentrations, transfusion history or severe pallor at admission, was a more common presentation, and consistent with earlier observations in Uganda [7] and recently reported in Kenya [31]. Those with SMA, while younger than patients presenting with cerebral malaria, include older children and this severe disease phenotype is not restricted to very young children, with 37.5% of those defined as SMA being 5–14 years of age. The observation of higher rates of SMA among older children requires further investigation and how to improve case-management in all pediatric age groups [51, 52]. Haemoglobinuria was reported as the presence of “tea colored urine” by caretakers among 4.8% of all malaria admissions (Table 3). The reported haemoglobinuria may be a part of the Blackwater fever syndrome, an increasingly common feature of severe malaria in parts of Uganda [53, 54], but interestingly might be less common outside Uganda [53].

We have described malaria admission among communities with relatively easy access to emergency care services in Uganda. The overall incidence of hospitalisation with malaria was 7 per 1000 children aged 1 month to 14 years of age per annum, ranging from very low levels in areas with very little transmission to over 20 per 1000 per annum in high transmission settings. These rates of hospitalized care for paediatric malaria are high and provide confirmatory evidence of the continued importance of malaria as a contributor to poor child survival in Uganda. Our estimates do not capture those with severe malaria who seek care elsewhere or die before reaching hospital. Treatment seeking for severe malaria remains poorly defined and requires further investigation, not just for the interpretation of hospitalised disease incidence but to also define points of improved pre-referral care [55–57]. Our results therefore represent approximate estimates of community acquired malaria requiring in-patient care. However, selecting catchments with relatively easy access to emergency care allows for a more reliable estimate of severe, potentially life-threatening disease incidence, compared to incidence defined among communities with limited access. A more comprehensive understanding of the epidemiology of severe, life-threatening disease and death requires prospective surveillance, however there are very few studies that have examined the long-term parasite exposure versus changing severe diseases profile in Africa [58].

The electronic AFI surveillance system operated at the sentinel hospitals introduced a standardized paediatric admission record system including universal malaria testing. This system provided opportunities to examine linked spatial and clinical data. Standardized admission record systems have been introduced in other settings, as part of routine health systems improvement tools [59–61]. Inevitably, the completeness and reliability of information available within hospital data systems depends upon resources and the intended objectives of surveillance programs. Hospital surveillance provides unique opportunities to monitor the changing clinical epidemiology of severe malaria and other febrile illnesses and with minimal investment could be implemented in a sustained form at national sentinel sites [62]. Investments are required for admission and data clerks, information and data systems and ensuring a basic minimum supply of laboratory supplies. These surveillance systems of severe disease we contend would be valuable resources to understand the changing landscape of severe disease epidemiology across Africa.

## Conclusion

Our results suggest that the epidemiology of severe, life-threatening malaria is consistent with early and frequent parasite exposure, and subsequent acquisition of immunity. In high transmission settings, under conditions of intense malaria control and reduced transmission for several years, the risk of severe malaria shifts to older children. This shift manifests in later years, as an increase in risk of severe malaria in older children, otherwise unexpected if transmission had not been interrupted. However, despite this increase, severe disease remains a rare event in children aged 10 years and above. The present analysis highlights the importance of both historical and current malaria exposure upon patterns of severe disease in a community. Current disease risks are determined by present exposure, but in the context of historical exposure and acquired immunity, complicating interpretation of observations based on ecological comparisons between sites over time. However, linkage of well-defined communities with easy access to hospital care provides better insight into the changing disease epidemiology resulting from changing transmission intensity. Indeed, longer-term hospital surveillance linked to the geography of hospital access, malaria control interventions, and parasite exposure offers future possibilities for better understanding of the epidemiology of severe malaria in Africa.

## Supplementary information

**Additional file 1: Supplement S1.** The incidence rate ratio (IRR) of age group adjusted for site obtained from a Poisson regression model.

**Additional file 2: Supplement S2.** Malaria admission rates for children aged 1 month-23 months per 1000 person-years of observation versus school-based malaria parasite prevalence at the end of the surveillance period.

## Data Availability

The datasets used and/or analysed during the current study are available from the corresponding author on reasonable request.

## Abbreviations

AFI: Acute Febrile Illness
CI: Confidence interval
DHIS-2: District Health Information System-2
IQR: Inter-quartile range
IR: Incidence rate
IRS: Indoor residual spraying
mRDT: Malaria Rapid Diagnostic Test
LLIN: Long-lasting insecticide treated nets
MRF: Medical Record Form
NMCP: National Malaria Control Programme
SMA: Severe malaria anaemia
UMSP: Uganda Malaria Surveillance Project
