# Supplementary material for: The age-specific incidence of hospitalized paediatric malaria in Uganda
Source: BMC Infect Dis. 2020 Jul 13;20:503. doi: 10.1186/s12879-020-05215-z (PMC7359223; doi:10.1186/s12879-020-05215-z)
Supplement: Supplementary file 1 — Additional file 1: Supplement S1. The incidence rate ratio (IRR) of age group adjusted for site obtained from a Poisson regression model. [file 12879_2020_5215_MOESM1_ESM.docx]

**Additional File: Supplement S1.** The incidence rate ratio (IRR) of age group adjusted for site obtained from a Poisson regression model

|  | **IRR** | **95% CI** | | **P-value** |
| --- | --- | --- | --- | --- |
| **Age group** |  |  |  |  |
| 1 – 4 years | 1.00 |  |  | <0.001 |
| 1 – 11 months | 0.97 | 0.84 | 1.13 |  |
| 5 – 9 years | 0.28 | 0.25 | 0.32 |  |
| 10 – 14 year | 0.09 | 0.07 | 0.11 |  |

**Foot note.** A Poisson regression was used to determine the association between age group and the rate of malaria hospitalisation, adjusting for site. Children aged 1 – 4 years were used as the reference age group as they had the highest of risk of malaria hospitalization and site was adjusted for because of the heterogeneity in the transmission levels between sites.
