# Supplementary figures and images for: The age-specific incidence of hospitalized paediatric malaria in Uganda
Source: BMC Infect Dis. 2020 Jul 13;20:503. doi: 10.1186/s12879-020-05215-z (PMC7359223; doi:10.1186/s12879-020-05215-z)

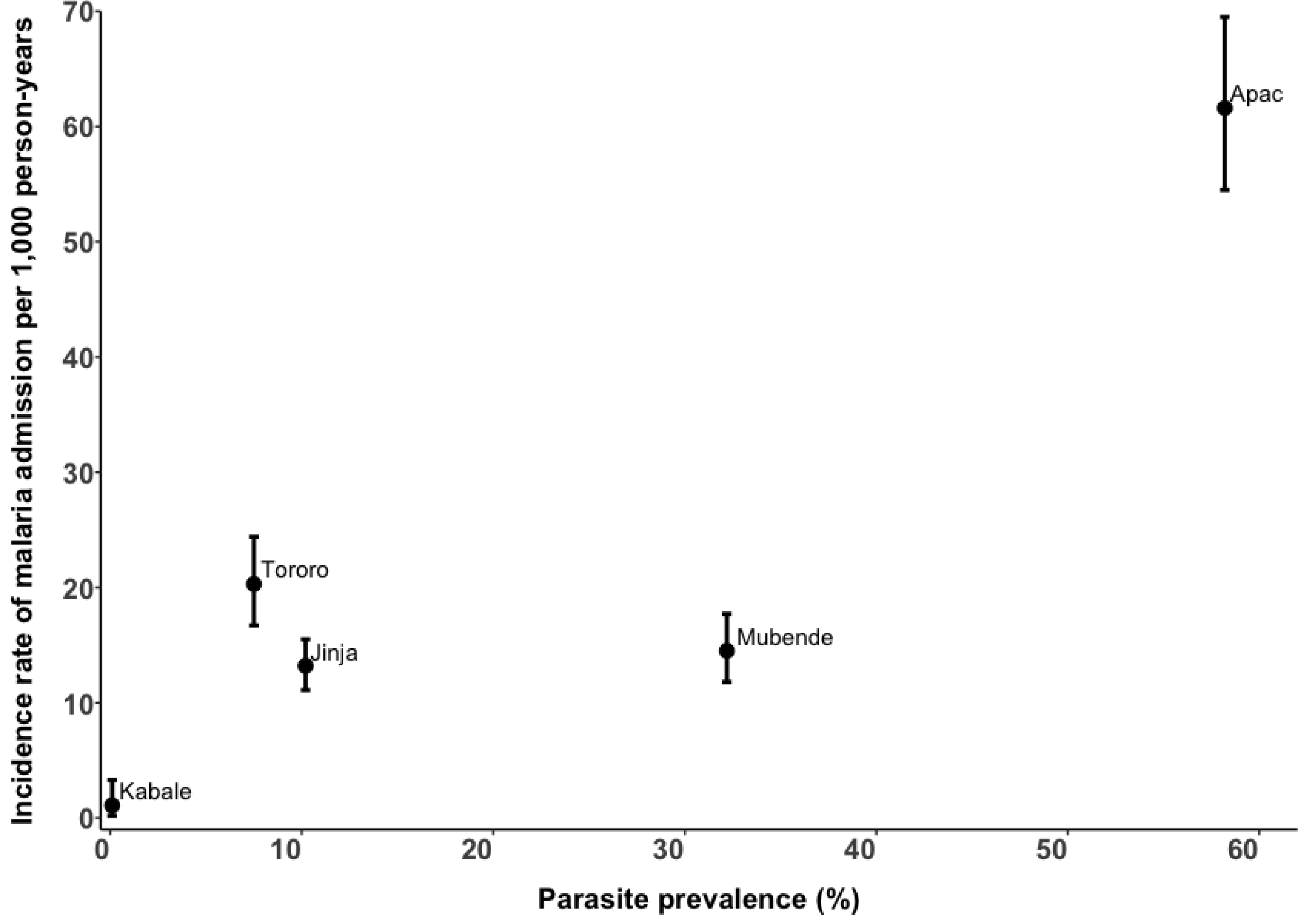

Supplement: Supplementary file 2 — Additional file 2: Supplement S2. Malaria admission rates for children aged 1 month-23 months per 1000 person-years of observation versus school-based malaria parasite prevalence at the end of the surveillance period. [file 12879_2020_5215_MOESM2_ESM.tif]
